# Supplementary material for: Effects of Soaking on the Volatile Compounds, Textural Property, Phytochemical Contents, and Antioxidant Capacity of Brown Rice
Source: Foods. 2022 Nov 18;11(22):3699. doi: 10.3390/foods11223699 (PMC9689972; doi:10.3390/foods11223699)
Supplement: Supplementary file 1 [file foods-11-03699-s001.zip › foods-1954779-supplementary.pdf]

Table S1 The texture properties of cooked brown rice under different soaking conditions

**Samples**	**Hardness (g)**	**Stickiness (g.sec**
Unsoaked rice	2033±84$_{a}$	176.5±29.7$_{f}$
	**Soaking at different conditions**	
40°C-30min	2001±96$_{ab}$	374.1±39.5$_{ab}$
40°C-60min	1975±95$_{ab}$	397.2±25.4$_{a}$
50°C-30min	1995±93$_{ab}$	305.0±27.6$_{cd}$
50°C-60min	1846±73$_{cd}$	387.0±36.4$_{a}$
60°C-30min	1916±75$_{bc}$	287.4±28.7$_{de}$
60°C-60min	1734±85$_{de}$	344.6±27.2$_{bc}$
70°C-30min	1816±78$_{cd}$	255.6±21.1$_{e}$
70°C-60min	1639±50$_{e}$	293.8±27.7$_{d}$

Means followed by different lowercase letters within rows are significantly different (p <0.05).
